# Assessing the INDCs’ land use, land use change, and forest emission projections

**DOI:** 10.1186/s13021-016-0068-3

**Published:** 2016-12-08

**Authors:** Nicklas Forsell, Olga Turkovska, Mykola Gusti, Michael Obersteiner, Michel den Elzen, Petr Havlik

**Affiliations:** 1 International Institute for Applied System Analysis (IIASA), Schlossplatz 1, 2361 Laxenburg, Austria; 2 Lviv Polytechnic National University, 12 Bandera Street, Lviv, 79013 Ukraine; 3 PBL Netherlands Environmental Assessment Agency, Bezuidenhoutseweg 30, 2594 AV The Hague, Netherlands

**Keywords:** LULUCF, INDCs, Paris Climate Agreement

## Abstract

**Background:**

In preparation for the 2015 international climate negotiations in Paris, Parties submitted Intended Nationally Determined Contributions (INDCs) to the United Nations Framework Convention on Climate Change (UNFCCC) expressing each countries’ respective post-2020 climate actions. In this paper we assess individual Parties’ expected reduction of emissions/removals from land use, land use change, and forest (LULUCF) sector for reaching their INDC target, and the aggregate global effect on the INDCs on the future development of emission and removals from the LULUCF sector. This has been done through analysis Parties’ official information concerning the role of LULUCF mitigation efforts for reaching INDC targets as presented in National Communications, Biennial Update Reports, and Additional file 1.

**Results:**

On the aggregate global level, the Parties themselves perceive that net LULUCF emissions will increase over time. Overall, the net LULUCF emissions are estimated to increase by 0.6 Gt CO_2_e year^−1^ (range: 0.1–1.1) in 2020 and 1.3 Gt CO_2_e year^−1^ (range: 0.7–2.1) in 2030, both compared to 2010 levels. On the other hand, the full implementation of the INDCs is estimated to lead to a reduction of net LULUCF emissions in 2030 compared to 2010 levels. It is estimated that if all conditional and unconditional INDCs are implemented, net LULUCF emissions would decrease by 0.5 Gt CO_2_e year^−1^ (range: 0.2–0.8) by 2020 and 0.9 Gt CO_2_e year^−1^ (range: 0.5–1.3) by 2030, both compared to 2010 levels. The largest absolute reductions of net LULUCF emissions (compared to 2010 levels) are expected from Indonesia and Brazil, followed by China and Ethiopia.

**Conclusions:**

The results highlights that countries are expecting a significant contribution from the LULUCF sector to meet their INDC mitigation targets. At the global level, the LULUCF sector is expected to contribute to as much as 20% of the full mitigation potential of all the conditional and unconditional INDC targets. However, large uncertainties still surround how Parties estimate, project and account for emissions and removals from the LULUCF sector. While INDCs represent a new source of land-use information, further information and updates of the INDCs will be required to reduce uncertainty of the LULUCF projections.

**Electronic supplementary material:**

The online version of this article (doi:10.1186/s13021-016-0068-3) contains supplementary material, which is available to authorized users.

## Background

Prior to the 2015 international climate negotiations in Paris, countries submitted INDCs to the United Nations Framework Convention on Climate Change (UNFCCC) [1]. The INDCs formed the core of mitigation commitments to be achieved within the context of the Paris Agreement by expressing each countries’ respective post-2020 emission reduction targets [2]. A total of 160 INDCs (187 countries, as the EU28^1^ submitted one INDC on behalf of its Member States) were submitted to the UNFCCC by December 15th, 2015 [1]. One of the core components of the INDCs was countries national target and actions for reducing greenhouse gas (GHG) emissions. Numerous countries put forward the targets and actions without any attached conditions (e.g. USA, Russian Federation, Canada). However, there are also countries that specified that part or all of their INDC is contingent on factors such as the availability of financial or technological support, actions taken by other countries, and the realization of international cooperation mechanisms (e.g. Indonesia, Mexico, Turkey).

A number of studies and reports have assessed the collective contribution of the INDCs in terms of reducing GHG emissions and prospects for limiting warming to 2 °C above preindustrial levels [e.g., 3, 4], as summarised in Rogelj et al. [5], which evolved from the UNEP [6] assessment. On the whole, these studies have shown that while the full implementation of the INDCs do not lead to emission projections by 2030 of similar magnitude as cost-optimal 2 °C scenarios (starting reductions in 2020), they will lead to a significant reduction of global GHG emissions compared to GHG projections in the absence of climate policies. Rogelj et al. [5] estimated that if all conditional and unconditional targets of the INDCs were to be met, global net GHG emissions would potentially be reduced by 11 Gt CO_2_e year^−1^ (range: 9–15) by 2030 in comparison to the IPCC AR5 no-policy baseline development from the IPCC AR5 Scenario Database.

One of the factors behind the range of estimated future emissions relates to differences in how countries consider the land-use sector in their INDCs [5]. Countries can choose whether to include the land-use sector or not, and can also select the accounting approach to be used. While emissions from the land use, land use change, and forest (LULUCF) are included for a majority of the INDCs, a number of countries either implicitly or explicitly exclude the land-use sector (e.g. Belarus, Egypt), and some countries indicate that a decision on whether to include LULUCF will be taken at a later stage (e.g. Republic of Korea). Countries can also use an accounting approach of their choice for their INDC and Parties have chosen to use different approaches. Amongst the Parties that specify an accounting approach for the LULUCF sector, a number of Parties have selected to use the net–net accounting approach (e.g. Canada, USA, Australia), while others have declared that they will use accounting approaches similar to those under the Kyoto Protocol (e.g. Japan, Switzerland). Unfortunately, numerous countries that include the land-use sector in their INDCs do not specify which accounting approach will be used, and the choice of an accounting approach can have substantial effect on the emissions of individual countries [7].

Whether or not Parties are considering the LULUCF sector as a component for reaching national targets as formulated in their INDCs, is valuable information for analyzing whether there are discrepancies between the scientific understanding of mitigation efforts needed to reach global targets and what mitigation options are being adopted by Parties in terms of domestic policies. In the scientific literature, it has been highlighted that while CO_2_ emissions from land use change account for as little as 6–17% of total current CO_2_ emissions [8], forests and the land use sector can play a key role in reducing emissions to the atmosphere, enhance the sequestration of carbon in terrestrial reservoirs, and substitute carbon intensive products. Houghton et al. [9] have shown how improvements in management of tropical forests could be used to stabilize atmospheric CO_2_ concentration during a transitional removal of fossil fuels. Establishment of forests on lands previous forested or non-forested areas has been put forward as a key strategy to increase the uptake of carbon from the atmosphere and increase biomass stocks [10–12]. However, it is recognised that these are also trade-off associated with these action as they can, on the one hand, lead to increased land use pressure [13], while at the same time can also lead to positive effects such as an improvement in biodiversity protection [14], restoration of degraded land [15], and improvements in water quality [16]. Reducing GHG emissions from deforestation and forest degradation (REDD) has also been put forward as a key strategy to conserve existing carbon pools in forest vegetation as well as providing a range of social, economic, and environmental benefits [17]. Reducing uncertainty concerning historical emissions from the land-use sector is also important [18], particularly reducing uncertainty concerning emissions from deforestation, forest degradation, forest carbon stocks, and peat land conversion.

While the INDCs have been estimated to lead to a significant reduction of the aggregate global GHG emissions, the role and contribution of the LULUCF sector within these commitments has not yet been fully analyzed and the contribution from individual Parties has not been clarified. The UNFCCC report [3] estimated that the INDCs would globally decrease the net LULUCF emissions by 1.1 Gt CO_2_e year^−1^ by 2030, relative to 2005 levels. The study was performed based on the emissions and removals related to the LULUCF sector, as stated by countries’ INDCs. However, as the UNFCCC report has a mandate to focus only on the aggregate effects, analysis of the contribution of individual Parties are not presented. It is therefore not possible to draw conclusions concerning which Parties that are considering the LULUCF sector as an important component for reaching national INDC targets. Furthermore, the study does not compare the aggregated effect of the INDCs to the global business-as-usual LULUCF projections, making it impossible to assess the importance of the INDCs for reaching the necessary global reduction of net LULUCF emissions.

In this study we assess individual Parties’ expected mitigation contribution from the LULUCF sector and the Parties that expect the largest contribution from the LULUCF sector for reaching their INDC target. Furthermore, we in this study analyze the overall global aggregate LULUCF mitigation estimate, as presented in the 160 Parties unconditional and conditional INDC targets. For this purpose, we evaluate the mitigation efforts within the INCDs that are directly related to the LULUCF sector by contrasting an INDC mitigation projection with a BAU projection. To reflect national developments as closely as possible, the BAU and INDC mitigation scenario developments are fully based on LULUCF estimates and projections provided in the Parties INDCs (where provided) [1] and complemented with information provided in the National Communications [19], biennial update reports, or supporting documents (see Table 2 for an overview of data sources being used for individual countries). Overall, the BAU and INDC projections for the LULUCF sector were available for 27 countries, while for 21 countries only INDC development dynamics were found, and for 8 countries only BAU projections were accessible. If a Party reported no information about its emission pathways, the BAU and INDC projections were assumed to stay constant over time to only consider the countries that have provided information concerning their expected development of the LULUCF emissions (see Table 2).

## Results

### Overview of LULUCF in the INDCs

To assess the importance that Parties placed on the LULUCF sector, we first assessed the extent to which Parties included emissions and removals from the LULUCF sector within their INDCs. The 160 Parties considered in this study, in total, contribute to about 98% of the net global land use emissions in 2010 of about 3 Gt CO_2_e year^−1^ according to FAOSTAT data [20].

Overall, the Parties appear very conservative in establishing LULUCF sector targets as well as in quantifying the reduction effect of future LULUCF measures. Of the 160 INDCs that have been assessed, 106 Parties explicitly state that emissions and removals from the LULUCF sector are included in the mitigation component of their INDC (see Table 1, two left columns). However, only 38 of these 106 Parties provide quantifiable details of measures or specific targets for the LULUCF sector. This group of Parties contributes to about 76% of net global land use emissions in 2010. Some Parties provide information on the development of net LULUCF emissions over time in BAU and INDC mitigation scenarios (e.g. Madagascar, Mali), or refer to a complementary report where such information can be found (e.g. Brazil, Indonesia, South Africa). Other Parties only provide estimated LULUCF emission levels based on the effect of proposed general reduction measures. Many Parties provide estimates of LULUCF emission reductions based on measures and policies specifically related to the LULUCF sectors (e.g. Japan, Guyana). Some Parties provide information about the area that will be afforested or the amount of carbon that will be sequestered as a result of improvements in forest management or build-up of the forest carbon stock (e.g. China, India).

**Table 1:** Categorization of Parties according to whether the LULUCF sector is covered in the submitted INDCs

LULUCF is covered and measures and/or specific targets are explicit	LULUCF is covered but no measures and/or specific targets are listed	LULUCF is partly covered	LULUCF is not covered
Algeria, Angola, Australia, Azerbaijan, Benin, Bolivia, Brazil, Burundi, Cabo Verde, Cambodia, Central African Republic, Chad, China, Comoros, R of Congo, DR Congo, Eritrea, Ethiopia, Gabon, Ghana, Guyana, Haiti, India, Indonesia, Japan, Lesotho, Madagascar, Malawi, Mali, Morocco, Namibia, Norway, Senegal, South Africa, Sudan, Uganda, Uruguay, Zambia	Afghanistan, Antigua and Barbuda, Argentina, Armenia, Bahamas, Belize, Bhutan, Bosnia-Herzegovina, Brunei Darussalam, Burkina Faso, Canada, Colombia, Costa Rica, Djibouti, Dominica, Dominican Republic, Ecuador, El Salvador, Equatorial Guinea, Grenada, Guatemala, Guinea Bissau, Iceland, Jordan, Kazakhstan, Kenya, Kiribati, Kyrgyzstan, Lao People’s Democratic Republic, Lichtenstein, Malaysia, Mauritania, Mauritius, Mexico, Mozambique, Myanmar, New Zealand, Niger, Nigeria, Papua New Guinea, Paraguay, Peru, Philippines, Rep. of Moldova, Russia, Rwanda, Saint Vincent and the Grenadines, San Marino, Sao Tome and Principe, Serbia, Sierra Leone, Singapore, Solomon Islands, Somalia, South Sudan, Sri Lanka, Suriname, Switzerland, Tajikistan, Tanzania, Togo, Tunisia, Turkey, Ukraine, United States, Vanuatu, Venezuela (Bolivarian Republic of), Vietnam	Bangladesh, Cameroon, Chile, Côte d’Ivoire, EU28 Member States, Gambia, Georgia, Guinea, Lebanon, Liberia, Mongolia, Samoa, Thailand, Tonga, Zimbabwe	Albania, Andorra, Bahrain, Barbados, Belarus, Botswana, Cook Islands, Cuba, Egypt, Fiji, Honduras, Israel, Iran (Islamic Republic of), Iraq, Jamaica, Kuwait, Maldives, Marshall Islands, Micronesia (Federated States of), Monaco, Montenegro, Nauru, Niue, Oman, Pakistan, Palau, Qatar, Rep. of Macedonia, Saint Kitts and Nevis, Saint Lucia, Saudi Arabia, Seychelles, South Korea, Swaziland, Trinidad and Tobago, Turkmenistan, Tuvalu, United Arab Emirates, Yemen
38 parties	68 parties	42 Parties	39 parties
76.2% of global net LULUCF emissions in 2010	25.6% of global net LULUCF emissions in 2010	−4.2% of global net LULUCF emissions in 2010	0.4% of global net LULUCF emissions in 2010

The share of global net LULUCF emissions is calculated from [20]

The remaining 68 Parties state in their INDCs that the LULUCF sector is covered in their mitigation targets, without providing LULUCF projections or quantifiable information concerning LULUCF mitigation policies. Based on FAOSTAT data, the contribution of this group to net global land use emissions in 2010 is estimated to be 25.6%. For instance, some Parties provide a list of measures and policies in the LULUCF sector but do not include the data needed for estimating LULUCF emission reductions (e.g. Jordan). Other Parties state that the LULUCF sector is covered but do not specify a LULUCF reduction target or mitigation measures (e.g. Russia, New Zealand, United States).

This leaves 54 Parties who explicitly state that the LULUCF sector is not included in their INDC mitigation targets. In this group, 15 Parties, which contribute to −4.2% of global net LULUCF emissions in 2010, nevertheless do propose measures or policies related to the LULUCF sector (e.g. Chile, Georgia), with some of them stating that the decision whether or not to include the LULUCF sector in mitigation targets will be taken by 2020 (e.g. EU28, Thailand). The remaining 39 Parties that cover less than 1% of net global land use emissions in 2010 state that the LULUCF sector is not covered and also do not propose measures or policies for reducing LULUCF emissions. Some of these Parties do not mention the LULUCF sector at all (e.g. Moldova, Andorra), or only mention the possibility that the LULUCF sector will be included at a later stage (e.g. Republic of Korea, Montenegro).

### National business-as-usual scenarios for the LULUCF sector

National BAU scenarios were created based on the information made available by Parties concerning their future trends in LULUCF emissions and removals. The national BAU scenarios were created based on official data sources provided by the Parties in their INDCs and complemented with information from National Communications, GHG inventories [21], and Additional file 1 (see Table 2). For Parties that did not explicitly state that emissions and removals from the LULUCF sector are included in the mitigation component of their INDC, net LULUCF emissions were assumed to stay constant over time according to the last available historical data points. In order to arrive at total global emissions estimates and provide comparability with other estimates, global net LULUCF emissions were harmonized to historical GHG emissions estimates [20] through the use of a harmonization factor that stays constant over time. More detailed information about particular countries and further explanations about the harmonization procedure can be found in the “Methods” section.

**Table 2:** Information sources used for projecting net LULUCF emissions in national BAU and INDC scenarios, for the 95 countries whose INDCs explicitly state that the LULUCF sector is covered

LULUCF projections in both the BAU and INDC scenario are based on information from the INDC [1]	LULUCF projections in the BAU are based on information from the National Communication [19]; in the INDC scenario, on information from the INDC	LULUCF projections in both the BAU and INDC scenario are based on information from the National Communication	LULUCF projections in both the BAU and INDC scenario are based on information from supporting documents
Central African Republic^a^, Comoros, R of Congo, DR Congo, Ethiopia, Gabon, Madagascar, Mali, Senegal, Uganda	Algeria^a^, Angola^a^, Australia, Benin, Bolivia^a^, Burundi^a^, Cabo Verde^a^, Cambodia^a^, China^a^, Eritrea^a^, Ghana^a^, Guyana^a^, Haiti^a^, Japan^a^, Lesotho^a^, Malawi^a^, Morocco, Namibia^a^, Norway^b^, Sudan^a^, Uruguay^a^, Zambia^a^	Argentina^ab^, Afghanistan^ab^, Antigua and Barbuda^ab^, Armenia, Bahamas^ab^, Azerbaijan^ab^, Belize^ab^, Bhutan^ab^, Bosnia-Herzegovina, Brunei Darussalam^ab^, Burkina Faso^ab^, Canada^b^, Chad^ab^, Colombia^b^, Costa Rica^b^, Djibouti^ab^, Dominica^ab^, Dominican Republic^ab^, Ecuador^b^, El Salvador^ab^, Equatorial Guinea^ab^, Grenada^ab^, Guatemala^ab^, Guinea Bissau^ab^, Iceland^ab^, Jordan^b^, Kazakhstan^ab^, Kenya^ab^, Kiribati^ab^, Kyrgyzstan^ab^, Lao People’s Democratic Republic^ab^, Lichtenstein^ab^, Malaysia, Mauritania^ab^, Mauritius^ab^, Mexico, Mozambique^ab^, Myanmar^a^, New Zealand, Niger, Nigeria^ab^, Papua New Guinea^ab^, Paraguay^ab^, Peru, Philippines^ab^, Rep. of Moldova, Russia, Rwanda, Saint Vincent and the Grenadines^ab^, San Marino^ab^, Sao Tome and Principe^ab^, Serbia^ab^, Sierra Leone^ab^, Singapore^ab^, Solomon Islands^ab^, Somalia^ab^, South Sudan^ab^, Sri Lanka^ab^, Suriname^ab^, Switzerland, Tajikistan^ab^, Tanzania^ab^, Togo^ab^, Tunisia, Turkey^b^, Ukraine^b^, United States, Vanuatu^ab^, Venezuela (Bolivarian Republic of)^ab^, Vietnam	Brazil, India^a^, Indonesia, South Africa
10 Parties	22 Parties	70 Parties	4 Parties

^a^net LULUCF emissions in the national *BAU* scenario is kept constant over time according to the most recent historically available reporting period

^b^National *INDC* mitigation scenario is kept constant over time according to the most recent historically available reporting period

**Fig. 1:**
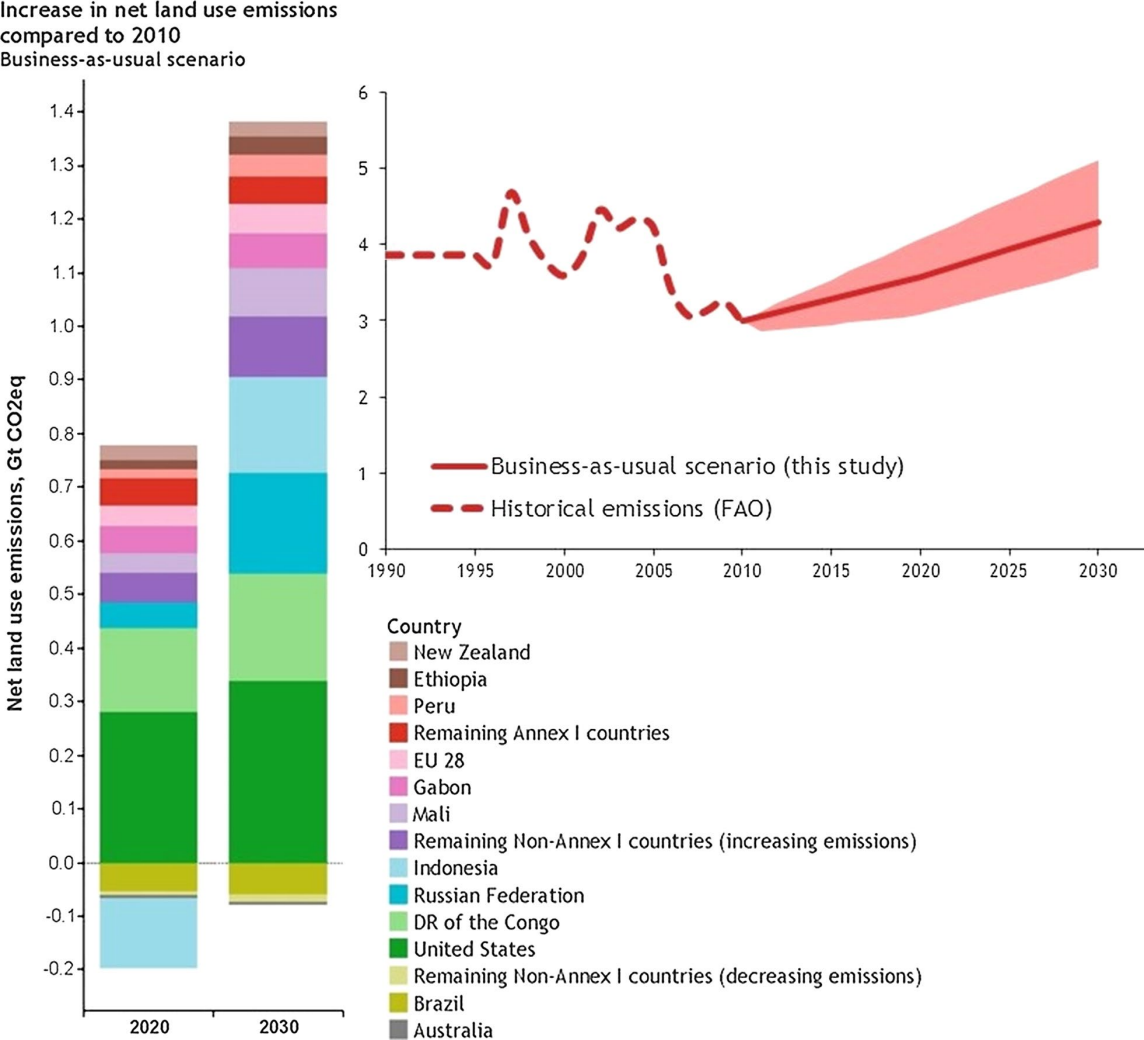
National business-as-usual projections of Parties emissions and removals from LULUCF

On the aggregate global level, it can be seen that the Parties perceive that the net LULUCF emissions will increase over time in comparison to 2010 levels (see Fig. 1). Overall, the increase in net LULUCF emissions is estimated to be 0.58 Gt CO_2_e year^−1^ (range: 0.1–1.1) by 2020 and 1.3 Gt CO_2_e year^−1^ (range: 0.7–2.1) by 2030, both compared to reported estimates for 2010 [20]. Of the Parties that have been assessed, a total of 26 have a national BAU development, where the net LULUCF emissions are projected to increase over time in comparison to 2010 levels. The substantial increase in net emissions from the LULUCF sector is projected for the DR Congo, Indonesia, USA, and the Russian Federation. Together, these four countries account for about 70% of the total global projected increase in net LULUCF emissions by 2030. Only Brazil has been assessed to have a national BAU development where the net LULUCF emissions are expected to significantly decreasing over time [22]. This decrease is strongly related to a slowdown of the deforestation rate in the Amazon. Indonesia also expects a decrease in net land use emissions as of 2020 (comparison to 2010 levels), mainly related to a decrease in peat fire and oxidation emissions [23]. However, in 2030 Indonesia expects to increase emission levels from land use and becomes one of the countries that contributes the most to the emissions increase according to the BAU development. Countries like Australia, Chile, Uganda, Republic of Congo, Morocco, Costa Rica, Armenia, Mexico, Tunisia, and Moldova individually show a slight decrease of net LULUCF emissions by 2030 in the range of 1–4 Mt CO_2_e year^−1^ compared to 2010 levels.

To provide further insights to the national developments, a range in emission projections was estimated. Uncertainty of future levels of net LULUCF emissions arises from issues such as ambiguity concerning accounting rules and methods to be considered by a Party, complicacy of predicting the effect of natural disturbances, definition of baseline, etc. For this work we focus on the uncertainty related to Parties that have not provided fully quantifiable information for land use emissions and removals pathways, as well as ambiguity among the reported sources of emission reduction and improvement of sinks. Assumptions taken for individual countries concerning uncertainty ranges are provided in the Methods section and Additional file 1. The uncertainty surrounding the national BAU development is recognized to be substantial. The BAU scenarios projects an increase of net LULUCF emissions to 3.6 Gt CO_2_e year^−1^ (range: 3.1–4.1) in 2020 and to 4.3 Gt CO_2_e year^−1^ (range: 3.7–5.1) in 2030. The largest uncertainty comes from Brazil, USA, Gabon, and the Russian Federation because the reference values (from which the reductions would be measured) and/or the BAU projections for the net LULUCF emissions are not explicitly stated in the INDCs of these countries.

### National INDC mitigation scenario for the LULUCF sector

The national INDC mitigation scenarios were created based on official data sources provided by the Parties in their INDCs and, where needed, complemented with LULUCF related emission reduction measures from National Communications, and Additional file 1.

**Fig. 2:**
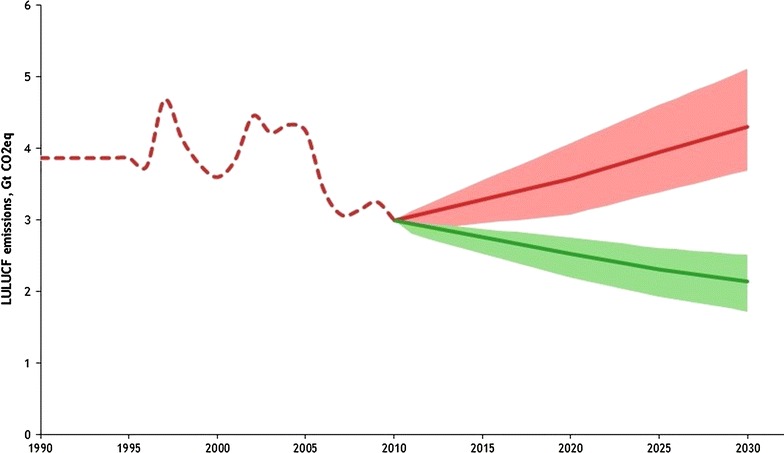
Business-as-usual and INDC projections of global net LULUCF emissions. Net LULUCF emissions expected to result from INDC implementation, compared to national business-as-usual projections based on official national data as provided in the INDCs [1] and National Communications [2]

The full implementation of the INDCs is estimated to lead to a reduction of net LULUCF emissions in 2030 compared to 2010 levels (see Fig. 2). We estimate that if all conditional and unconditional INDCs are implemented, net LULUCF emissions would decrease by 0.5 Gt CO_2_e year^−1^ (range: 0.2–0.8) by 2020 and 0.9 Gt CO_2_e year^−1^ (range: 0.5–1.3) by 2030, both compared to reported estimates for 2010 [20]. The projected global net emissions from the LULUCF sector in 2030 is approximately 2.1 Gt CO_2_e year^−1^ (range: 1.7–2.5) if all conditional and unconditional INDCs are implemented. The expected emission reduction of the INDCs mitigation scenarios are thus well below the increase in LULUCF as of the national BAU scenarios. Full implementation of the INDCs would decrease net LULUCF emissions in 2030 of 2.2 Gt CO_2_e year^−1^ (range: 2.0–2.6) compared to the national BAU development.

**Fig. 3:**
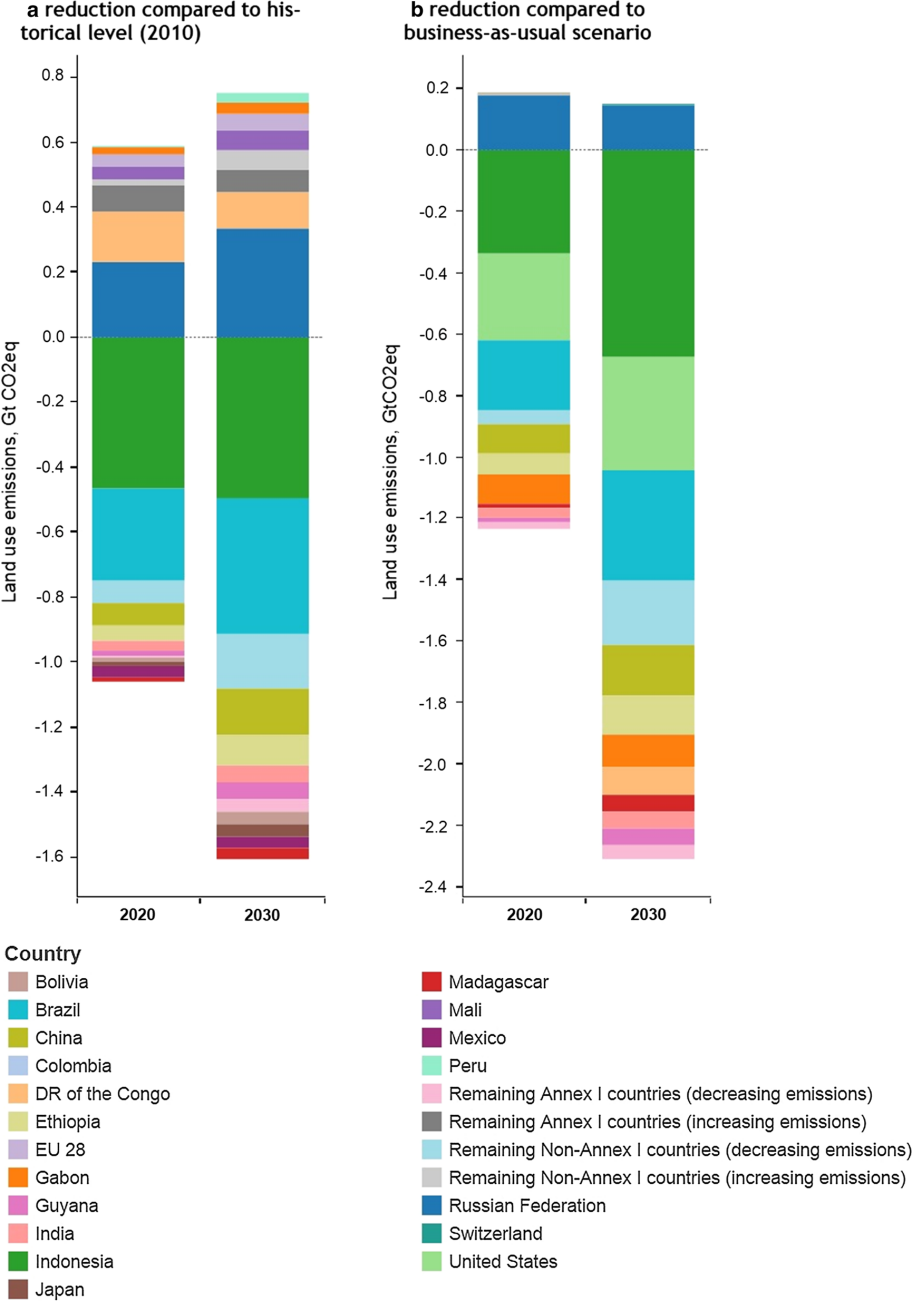
Impact of INDC on net LULUCF emissions compared to (**a**) business-as-usual projections, (**b**) historical 2010 levels. The countries which show the biggest reduction/increase of LULUCF emissions and removals in comparison to (**a**) business-as-usual projections, (**b**) historical 2010 levels. *Positive numbers* indicate emission reductions, *negative numbers* indicate emission increases

The analysis of the INDCs shows that the largest absolute reductions of net LULUCF emissions (compared to the BAU scenario development) are expected from Indonesia and United States, followed by Brazil, China, Ethiopia, Gabon and the DR Congo (see Fig. 3 and Table 3). The INDC for Indonesia indicates that a lion’s share of emission savings is expected from a reduction of the deforestation rate and reduced emissions from peat oxidation. Brazil is expecting a significant reduction of net LULUCF emissions through implementation of the Forest Code and achieving zero illegal deforestation in the Amazon biome. In the case of USA, net LULUCF emissions are expected to be reduced through maintenance of the current level of carbon sequestration and thereby avoid the expected loss of the carbon sink as projected in the BAU scenario. China is planning to reduce net LULUCF emissions through afforestation measures and enhancements of the national forest carbon stock. According to the INDC of Gabon, net emissions reductions (compared to the BAU scenario) will be reached through successful implementation of a number of mitigation policies relating to the forest sector (e.g., Code Forestier, Plan National d’Affectation de Terre). Reduction of net LULUCF emissions in Ethiopia is expected through an increase of the forest carbon stock, forest land protection and forest reestablishment. The INDC of DR Congo states that the reduction in net LULUCF emissions will mainly be achieved through the implementation of afforestation and reforestation measures.

**Table 3:** Historical, BAU and INDC mitigation levels of land use emissions for selected Party, Mt CO_2_e year^−1^

Party	2010	2020		2030	
	Historical level	BAU	INDC mitigation	BAU	INDC mitigation
Australia	16	12 (range: 8–12)	13	12 (range: 8–12)	11
Bolivia	38	38	25	38	−1
Brazil	402	347 (range: 347–730)	119 (range: 119–340)	342 (range: 342–1050)	−15 (range: −15 to 270)
China	−421	−421	−517 (range: −500 to −517)	−421	−586 (range: −500 to −586)
Colombia	26	26	27	26	28
DR of the Congo	190	345	345	390	300
Ethiopia	55	73	8	90	−40
EU 28	−294	−255	−256	−239	−242
Gabon	100 (range: 30 to 100)	150 (range: −60 to 150)	55 (range: −60 to 55)	165 (range: −60 to 165)	63 (range: −60 to 63)
Guyana	−55	−55	−72	−55	−107
India	−175	−175	−206	−175	−228
Indonesia	898	767	431	1075	403
Japan	−69	−69	−81	−69	−106
Madagascar	−220	−216	−231	−194	−253
Mali	−216	−179	−179	−127	−153
Mexico	47	46	11	46 (range: 45–46)	11 (range: −2 to 11)
Peru	43	58	48	85	71
Remaining Annex I countries	−372	−285	−268	−302	−280
Remaining Non-Annex I countries	265	302	187	348	125
Russian Federation	−651	−601 (range: −601 to −418)	−418 (range: −601 to −418)	−462 (range: −462 to −315)	−315 (range: −462 to −315)
Switzerland	−2	1	2	1	3
United States	−851	−569 (range: −853 to −569)	−853 (range: −853 to −569)	−512 (range: −884 to −512)	−884 (range: −884 to −512)

Uncertainties concerning levels of net LULUCF emissions for the INDC development were created in the same way as the uncertainty range concerning the BAU development (see the Methods section and Additional file 1 for further details concerning assumptions taken for individual countries). As such, we focus here on the uncertainties concerning the current level of fulfillment of emission reduction targets that relate to historical base year or reference values (e.g., Australia, China, Gabon), unclear scenario projections (e.g., Brazil, Mexico, the Russian Federation), as well as unclear sectorial composition of overall emission reduction targets (e.g. USA).

The uncertainties range for the INDC mitigation scenario as well as for the BAU scenario indicates almost equal possibility to increase and decrease the net land use emissions development. The highest uncertainties are related to developments in Brazil, Gabon, USA, and the Russian Federation. The other countries demonstrate relatively low uncertainties due to vague baseline and projections definitions in the land use sector. There were additional uncertainties that remained unquantified as they are beyond the scope of this study.

Despite, aggregately, the INDCs are globally projected to decrease net LULUCF emissions in 2030 compared to the 2010 level, there are Parties where the net LULUCF emissions will still increase over time, regardless of the mitigation effort of the INDC. In other words, for certain countries the fulfillment of the INDCs will not be sufficient to reduce net LULUCF emissions compared to a historical reference period. For Gabon, DR Congo, and Mali, the increase in net LULUCF emissions in the BAU developments is higher than the reduction of net LULUCF emissions through the full implementation of the INDCs. Also, for the Russian Federation and, to a lesser extent, Switzerland, Colombia, and Australia (only until 2020), the INDCs are expected to result in an increase in net LULUCF emissions. The impact of these increased net LULUCF emissions on INDC targets for overall emission reduction is highly dependent on the way in which changes in LULUCF emissions will be accounted for. For example, the projected increase in the Russian Federation’s net LULUCF emissions of approximately 150 Mt CO_2_e year^−1^ in 2030 (compared to 2010 levels) is directly related to intensification of forest management. More details on Party-level LULUCF reductions are provided in the “Methods” section.

### The importance of the LULUCF sector for the full achievement of the INDCs

Having quantified the LULUCF implication of the INDCs, we have been able to assess the importance of the land-use-related emissions within the context of the INDCs. To assess the importance of the LULUCF sector within the INDCs, the estimated emission reduction from the LULUCF sector was compared with the total cross-sectorial GHG emission reduction potential of the INDCs. Since within this study we have not assessed what the submitted INDCs would deliver in terms of total cross-sectorial GHG emission reduction, the quantified contribution of the LULUCF sector has to be compared with estimates from other studies.

Three studies of the aggregate emission impacts of the INDCs have been published with similar concluding estimates of the contribution of the conditional and unconditional INDC targets. The UNEP report [6] estimated that if all conditional and unconditional INDC targets were to be met, global net GHG emissions would potentially be reduced by 11 Gt CO_2_e year^−1^ (range: 8–13) in 2030 in comparison to the IPCC AR5 BAU scenario estimate (in absence of a climate policy). The updated analysis of Rogelj et al. [5], including new model studies such as [3] find a similar estimate. Furthermore, individual studies of den Elzen et al. [24] also discern with a similar reduction compared to a no policy baseline scenario.

Assuming that the INDCs have the potential to reduce global net GHG emissions in the range of 11 Gt CO_2_e year^−1^, it can be estimated that on the aggregate global level, the LULUCF sector is expected to contribute to roughly 20% of the full mitigation potential of all the conditional and unconditional INDC targets. However, it should be noted that this estimate is uncertain, as the national BAU LULUCF emission development applied in this study are not necessarily fully consistent with the no-policy baseline scenario as assumed in [5, 6, 24]. This inconsistency may be related to differences in data sources used for evaluating the INDCs, construction of baselines scenarios, impacts of mitigation actions, and differences in the INDCs that are accounted for in the studies.

## Conclusions

This study has reviewed the expected outcome of the LULUCF related measures as put forward by Parties’ in their INDCs ahead of the 2015 international climate negotiations in Paris. Using LULUCF information provided by the Parties, this study highlights that Parties are expecting a significant contribution from LULUCF in meeting the individually proposed INDCs mitigation targets. As much as 20% of the total greenhouse gas mitigation of the INDCs has been analyzed to be related to the LULUCF sector. A wide range of LULUCF mitigation options are being put forward by the Parties to reduce emissions and increase removals from the LULUCF sector. Options such as reducing deforestation, increasing afforestation, improving sustainable forest management, and enhancements of forest carbon stock are mentioned by the Parties INDCs. Other options such as the use of forest products to substitute carbon intensive material (i.e. material substitution effects), and the use of wood to substitute carbon intensive fossil fuels for energy production (i.e. energy substitution effects) are on the other hand not commonly described nor quantified within the INDCs and could be given greater consideration by the Parties. However, care would be required when deliberating such estimates that double counting of material and energy substitution effects does not occur and that clear estimates are provided. It should also be noted that a number of Parties have provided joint commitments for the LULUCF and agriculture sector (e.g. Mauritania, Namibia). As these two sectors highly interlinked, Parties have to carefully consider cross-sectorial implications when implementing mitigation options as well as develop projections that are consistent and feasible for both the sectors.

According to our INDC analysis, a relatively small set of countries—Indonesia, United States, Brazil, China, Ethiopia, Gabon and the Democratic Republic of Congo—have provided the lion share of pledges to reduce net LULUCF emissions. Together, these Parties account for about 84% of the total global expected reduction of net LULUCF emissions by 2030. If the ambitions of these Parties are achieved, reductions in the range of 1888 Mt CO_2_e year^−1^ would be achieved by 2030. There are still large methodological and technical differences between the LULUCF assessments provided by Parties. Consistency of assessments leading to improved comparability of Parties’ efforts to fulfillment of the reduction targets is still a challenge. Implementability and effectiveness of proposed LULUCF measures is also still difficult to assess mostly for a lack of detailed technical information.

While the exact estimate of the contribution from the LULUCF sector to the full mitigation potential of the INDC’s is surrounded with considerable uncertainty, the analysis has shown that the Parties themselves expected the LULUCF sector to play an important role in reaching the proposed INDC mitigation targets. A majority of Parties that have been analyzed treat the land-use sector as fully fungible to other sectors. Some countries have included the land-use sector but not decided how it will be included. There are also countries that include separate targets for the sector or do not include the land-use sector at all.

Implementation of policies and measures in the LULUCF sector will require a range of activities, which finally need to be qualified as additional in order to be compensated by international finance [25]. However, in the international negotiations under the UNFCCC, no mechanism is currently envisaged to promote distinctive “high quality LULUCF credits” that potentially also ensure multiple benefits (i.e. protection of biodiversity and other ecosystem services). Currently, as the INDCs demonstrate, there is a lack of technical know-how and capacity on issues that will ensure the additionality and environmental integrity of LULUCF measures. There is a pressing need to support countries at different stages of their planning process. This includes assisting countries in undertaking initial spatial analyses, establishing globally consistent national reference scenarios, and carry out LULUCF policy impact assessments accounting for altered carbon flows and indirect land use effects associated with changes in international trade [26].

We suggest that integrated land-use modelling will be key to support the design of globally consistent national and regional LULUCF policies. Such modelling effort will necessitate a global forum for sharing and improving global data, developing best practices and technical guidance for national policy modelling. Such a forum will support bilateral and multilateral efforts to ensure transparency, as well as the environmental and financial integrity of efforts in the LULUCF sector. Furthermore, such modelling efforts would help build national capacity for integrated planning to design policies for the agriculture, forestry, nature conservation and bio-energy sectors in an economy-wide and globally consistent way.

## Methods

### Calculation of LULUCF emissions and removals for the projections

Projections of LULUCF emissions and removals were created for each of the 106 parties that explicitly state in their INDC submission that the LULUCF sector is covered (see Table 1, two left columns). For these Parties, net LULUCF emissions were projected according to a national BAU scenario and a national INDC mitigation scenario in line with a full implementation of the INDC mitigation options.

The LULUCF projections are based on estimates provided in the national INDCs (where available) and/or projections and estimates presented in the National Communications or in supporting documents as officially provided (data sources being used for key countries are reported in the following subchapter). Data sources used for projections are summarized in Table 2, and an overview of land use projections for countries showing the largest increase and decrease in net LULUCF emissions is provided in Table 3. Where insufficient information was available to estimate LULUCF development over time (either in the BAU or INDC mitigation scenario), it was conservatively assumed that LULUCF emissions and removals would stay constant over time (e.g. Argentina, Canada, Kazakhstan, etc.). The countries that have not officially provided an LULUCF projection thereby do not influence the development of the aggregate global net LULUCF emissions over time.

### Selected countries’ contributions and construction of national scenarios

The INDC of *Brazil* covers the LULUCF sector but does not provide LULUCF projections corresponding to a BAU or INDC mitigation scenario. However, the INDC does contain a list of mitigation measures that specifically address the LULUCF sector (e.g. zero illegal deforestation in Brazilian Amazonia by 2030). The INDC reduction target for the LULUCF sector is considered as unconditional. Our estimate of the INDC mitigation potential is based on the recent REDD-PAC project report [22] which provides a BAU projection and a scenario with mitigation measures for the LULUCF sector that are comparable with the Brazilian INDC. To the extent of our knowledge, the projections provided in the REDD-PAC report are consistent with the INDC submission. For this study, the LULUCF projections presented in the REDD-PAC project report have been harmonized for the year 2010 according updated inventory reporting as presented in the First Biennial update report of Brazil [27], taking into account the same pools and sources of emissions and sinks.

According to the REDD-PAC report, the BAU scenario projects a continuation of the 2000 land use trend, including illegal deforestation in line with historical references and no implementation of forest restoration measures. Overall, the scenario is expected to lead to a continuation of the loss of forest cover, in particular a high conversion rate of unprotected mature forests areas to grasslands or pastures. No forest regrowth measures are considered in the scenario [25]. Given these developments, the BAU scenario projects net LULUCF emissions level for Brazil in the range of 347 Mt CO_2_e year^−1^ in 2020, and 342 Mt CO_2_e year^−1^ in 2030.

The INDC mitigation scenario for this study has been specified in line with the “Forest Code scenario” as provided by the REDD-PAC report [22]. The scenario assumes full implementation of the Forest Code, zero illegal deforestation within the Amazon biome, establishment of Legal Reserves, Small Farms Amnesty, the establishment of Environmental Reserve Tradable Certificates, and compulsory forest restoration after 2020 [25]. Overall, the Legal Reserve provision sets the minimum percentage of forest or native vegetation to be preserved for each rural property, the amnesty of small farms exempts landowners from the need to recover legal reserves in small properties, and the Environmental Reserve Tradable Certificates creates a legal venue to trade forest surplus certificates that can be used to offset a properties debt in legal reserves. The scenario is expected to lead to a comprehensive reduction of the annual deforestation rate and reduced loss of unprotected mature forests, resulting in an overall increase of the national forest cover by 32 million ha by 2030. These trends are expected to reduce emissions and increase the sink capacity of the LULUCF sector, resulting in net LULUCF emissions of about 119 Mt CO_2_e year^−1^ in 2020, and −15 Mt CO_2_e year^−1^ in 2030. Full implementation of Brazil’s INDC is thus estimated to reduce net LULUCF emission by 228 Mt CO_2_e year^−1^ in 2020 and 357 Mt CO_2_e year^−1^ in 2030, compared to the BAU scenario development. If the full emission reduction potential of the INDC measures would be achieved, the LULUCF sector would become a net sink of emissions in 2030 (removals from conservation units and indigenous lands are excluded).

As no BAU or INDC scenario projection was presented in the INDC, uncertainty ranges were formulated for Brazil. The upper uncertainty limit for the BAU scenarios is consistent with the BAU projections of the REDD-PAC report without harmonization of the projection to historical reporting of net LULUCF emissions from the first Biennial Update Report of Brazil [27]. The upper limitation of the BAU development thereby reaches 1050 Mt CO_2_e year^−1^ in 2030 [19]. No estimate with lower net LULUCF than that of the BAU scenario has been found.

The INDC of *China* does not provide LULUCF projections corresponding to a BAU scenario nor the INDC mitigation scenario, but does propose future mitigation measures aimed specifically at the LULUCF sector. The key target for the LULUCF sector as presented in the INDC is an increase of the national forest stock volume by 4.5 billion m^3^ in 2030, compared to the 2005 level. China’s target for reduction of net LULUCF emissions is unconditional. It can be noted that the increase of the national forest stock volume is an LULUCF related measure that is commonly referred to by China to mitigate climate change. Under the 2010 Copenhagen Accord [28], China committed to increase the national forest stock by 1.3 billion m^3^ and increase the forest area by 40 million ha by 2020. These target were also confirmed in the government strategy “Outlines on Promoting Ecological Civilization (2013–2020)” as published in 2015 [29]. The INDC of China reports that the target relating to the forest stock has already been achieved while further measures are required to reach the targeted increase of the forest area. The INDC stated that the forest carbon stock has increased by 2.28 billion m^3^ in 2014 (compared to 2005 levels), while the forest area has expanded by 21.6 million ha in 2014 (compared to 2005 levels).

As no BAU scenario has been provided by China in their INDC or in the 2nd National Communication [30], our assessment conservatively assumes a BAU scenario with constant net LULUCF emissions over time. The latest reported estimate of the national net LULUCF emissions level was found in the 2nd National Communication, which reports that the LULUCF sector is a net carbon sink at the level of −421 Mt CO_2_e year^−1^ for the year 2005. No estimate of the net LULUCF sink is provided in the 2nd National Communication after 2005 and no estimate is provided in the INDC.

The INDC mitigation scenario has been defined based on the INDC target of an increase of the national forest stock volume. The estimation of net LULUCF emissions level in 2030 is based on the assumption of a linearly increasing build-up of the forest area and biomass stock over the period of 2005 and 2030. In order to convert cubic meters of wood biomass into tons of carbon, a wood density factor of 0.5 t/m^3^ was applied as well as a carbon fraction for dry matter 0.5 based on IPCC Good Practice Guidance for LULUCF [31]. Based to the abovementioned assumptions, is it estimated that the enhancement of the forest carbon sink can save about 165 Mt CO_2_e year^−1^ in 2030 (compared to the BAU scenario). Consequently, net LULUCF emissions may reach −586 Mt CO_2_e year^−1^ in 2030.

In this study we also consider some uncertainties that arise from emission reduction targets of China that relate to historical reference periods. China indicated in the 2nd National Communication that they intend to increase the sink by 1.3 billion m^3^ compared to the level of 2005 [32] while in the INDC it is reported that there will be a growth of sink in 2014 by 2.28 billion m^3^ and a new target is set to increase the sink by 4.5 billon m^3^ compared to the level of 2005. It is not explicitly stated in China’s INDC how the new target is harmonized with the old one and/or the level of its fulfilment.

The INDC of *India* does cover the LULUCF and mitigation targets as presented for the LULUCF sector are considered as unconditional. However, the INDC does not provide LULUCF emission projections neither for the BAU scenario nor for the INDC mitigation scenario. The INDC does mention future mitigation measures in the LULUCF sector (e.g. afforestation) but no LULUCF projection for the BAU or INDC mitigation scenario are provided. No BAU scenario projection for the LULUCF sector are provided in the 2nd National Communication [33]. Therefore, our assessment conservatively assumes that BAU net LULUCF emissions stay constant over time, at the emission level of 2007 (−175 Mt CO_2_e year^−1^) as reported in India’s 2nd National Communication. No estimate of the net LULUCF sink is provided in the 2nd National Communication after 2007 and no estimate is provided in the INDC.

The INDC mitigation scenario for this study has been defined according to the 2014 assessment by the Planning Commission of the Government of India on the national potential for keeping economic growth within low-carbon development [34]. The report analysed the carbon sequestration potential of mitigation activities and major policies relating to the LULUCF sector. Three main categories of sequestration options were evaluated: (1) Conservation and Improvement of Existing Forests; (2) Afforestation, (3) Wood Products Use and Management. The contribution of the most known India’s policy for LULUCF sector—Green India Mission (GIM) is included amongst the afforestation directives.

In addition to the sequestration potential, the report also provides an LULUCF projection based on a partial implementation of the assessed mitigation options. The projection provided by the Planning Commission demonstrates that the most significant change of emissions and removals from the LULUCF sector relates to an increase of the forest land cover, and a reduction of emissions related to excessive harvest of firewood from degraded forest. Overall, enhancement of the forest carbon sink is expected to save 22.2 Mt CO_2_e year^−1^ by 2030, compared to 2007 estimates. Furthermore, improvements related to the collection of fuel wood are by 2030 expected to save an additional 27.84 Mt CO_2_e year^−1^, compared to 2007 levels. At the same time, net emissions from grassland in 2030 is expected to be increased by 1 Mt CO_2_e year^−1^ (compared to 2007 levels) mainly relating to afforestation activities reducing the total area of grassland. Net emissions and removals from croplands are expected to remain constant from 2007 until 2030.

Based on the assessment of mitigation options and projection described by the Planning Commission [34], it is thus estimated that the INDC mitigation scenario would lead to a development where the net LULUCF emissions level would be −206 Mt CO_2_e year^−1^ in 2020, and −228 Mt CO_2_e year^−1^ in 2030. Full implementation of India’s INDC is thus estimated to lead to reduction of net LULUCF emission by 29 Mt CO_2_e year^−1^ in 2020 and 51 MtCO_2_e year^−1^ in 2030, compared to the BAU scenario development.

The INDC of *Indonesia* covers the LULUCF sector within its overall unconditional target, but does not provide LULUCF projections corresponding to a BAU scenario nor an INDC mitigation scenario. However, LULUCF projections for both scenarios have been constructed based on publically available information in supporting documents [23]. To the extent of our knowledge, the projections provided in the supporting documents are consistent with the INDC submission. Our estimations on Indonesia include the AFOLU sector since ascertaining only the land use sector was not possible. It should be noted that the projections (for both scenarios) take into account emissions related to peat oxidation and peat fires. According to Indonesia’s INDC, the government will focus on mitigation actions such as ecosystem conservation and restoration, coastal zone protection, and reduction of forest degradation and deforestation. The BAU scenario presented in the supporting documents projects net AFOLU emissions level of about 767 Mt CO_2_e year^−1^ in 2020, and 1075 Mt CO_2_e year^−1^ in 2030. On the other hand, implementation of the INDC is estimated to lead to emission reductions of approximately 336 Mt CO_2_e year^−1^ in 2020 and 672 Mt CO_2_e year^−1^ in 2030, compared to the BAU scenario development.

It should be noted that historical net AFOLU emissions and projections for the AFOLU sector have been significantly revised in the supporting documents [23] as compared to earlier data presented in the 2nd National Communication [35]. Net AFOLU emissions for 2010 are estimated in the range of 1460 Mt CO_2_e year^−1^ in the supporting documents, while the 2nd National Communication provides estimates in the range of 2505 Mt CO_2_e year^−1^. The main source of difference between the estimates related to emissions due to peat oxidation and peat fires which are estimated in the range of 559 Mt CO_2_e year^−1^ in the supporting documentation, as compared to 1442 Mt CO_2_e year^−1^ in the 2nd National Communication. The BAU projections for Indonesia have also been significantly revised. The supporting documents has a BAU projection of net AFOLU emissions of about 768 Mt CO_2_e year^−1^ in 2020, and the supporting documents has a BAU projection with net AFOLUC emissions of about 1635 Mt CO_2_e year^−1^ in 2020.

The INDC of the *Russian Federation* explicitly states that the LULUCF sector is included in the mitigation component of the INDC. However, the INDC does not provide LULUCF projections for the BAU scenario or the INDC mitigation scenario. No BAU projection is provide in the 6th National Communication for the LULUCF sector as a whole, however, four national forest management scenarios are provided within the report. The scenarios reflect four different levels of forest management intensity and resulting forest carbon sequestration level for the period of 2010 and 2030. Projections of net LULUCF emissions have therefore based on the forest management intensification scenarios, as provided in the 6th National Communication of the Russian Federation [36]. According to the 6th National Communication, the LULUCF sector in the Russian Federation was a net carbon sink in 2010 at the level of −651 Mt CO_2_e year^−1^.

The BAU scenario for this study has been specified in line with national forest management scenario from 6th National Communication that assumes that forest management intensity will stay constant at current level (scenario number 1 in the 6th National Communication). This scenario is expected to lead to an overall reduction of the forest carbon sink over time, mainly relating to forests becoming more mature which decreases the overall sequestration capacity over time. In 2030, the scenario is expected to lead to a reduction of the carbon sink in managed forests by 188 Mt CO_2_e year^−1^ as compared to the 2010 level. It should be noted that according to the 6th National Communication, the scenario is based on the assumption that forest fires and forest regeneration activities are assumed to stay constant at current level.

The INDC mitigation scenario of this study has been specified in line with the national forest management scenario with the highest forest management rate (scenario number 4 in the 6th National Communication). The scenario foreseen that the annual harvest level will increase rapidly from 2010 onwards and that by 2020 the harvest level will reach the annual allowable cut as defined by the state. The annual allowable cut is a measure defined by the state representing the amount of wood that is allowed to harvest from managed and protected forest during a year [37]. No information is provided in the 6th National Communication concerning the development of the annual harvest level for the scenario after 2020. Relating to the expected increase of forest harvest, the carbon sink in managed forests is expected to be reduced by 335 Mt CO_2_e year^−1^ in 2030, as compared to 2010 levels. The INDC mitigation scenario is as such expected to results in a reduction of the forest carbon sink by 147 Mt CO_2_e year^−1^ in 2030, compared to the BAU scenario development.

As only projection for forest that will remain forest could be estimated based on the information provided in the 6th National Communication, it is assumed that emissions and removals from reporting categories as croplands, grasslands, wetlands and settlements will stay constant over time at the level of 2010 (40.2 Mt CO_2_e year^−1^) in the BAU and INDC scenarios. Furthermore, it has to be assumed that the carbon stock projections as described in the 6th National Communication constitutes net forest land emissions.

It is uncertain whether or not the scenarios provided in the 6th National Communication are consistent with INDC target of Russian Federation and/or to what extend they will be implemented. The forest management intensification scenarios as presented in the 6th National Communication cannot with full certainty be adopted as the BAU scenario for net LULUCF emission levels under the INDC target. It should be noted that scenario number 2 and 3 as presented in the 6th National Communication would lead to a lower reduction of the carbon sink in managed forest than that of scenario number 4 which is here used as the INDC mitigation scenario. Therefore, uncertainty ranges for the Russian Federation have thereby been created, taking into account the ambiguity concerning the BAU and INDC mitigation scenario projections. The uncertainties and lack of available data do not allow for any conclusion regarding the unconditional and conditional targets for the LULUCF sector of the Russian Federation.

The LULUCF sector of the *USA* has remained a net sink over the period of 1990–2011. According to the 6th National Communication [38], not only has the LULUCF carbon sink remained but by 2011 it had also been increased by 14% compared to 1990 levels. The INDC of the USA explicitly states that the LULUCF sector is covered within an overall unconditional emissions reduction target, but does not provide LULUCF projections corresponding to the BAU scenario or the INDC mitigation scenario. An emission reduction target for the AFOLU sector is provided by the US State Department of Agriculture in “Building blocks for Climate Smart Agriculture & Forestry” [39]. The strategy proposed in this document is to reduce net emissions and enhance carbon sequestration by over 120 Mt CO_2_e year^−1^ by 2025. However, the document does not specify potential reductions in the LULUCF sector. Some studies assume that potential saving within the LULUCF sector by the strategy can be around 60 Mt CO_2_e year^−1^ (half the reduction stated in the strategy [40]). In this study, potential reductions within the program are considered in terms of uncertainties for INDC development scenario.

Since the estimation of future emission levels in the LULUCF sector cannot be completed based on the INDC or supporting documents, the BAU and INDC mitigation scenarios have been specified based on LULUCF projections provided in the 6th National Communication. The 6th National Communication provides two scenarios (high sequestration and low sequestration scenarios) for LULUCF emission levels development by 2030 [38]. The scenarios are based on different assumptions of carbon sequestration pathways. In the high sequestration scenario, net LULUCF emissions will roughly remain at the current level, while in the low sequestration scenario the LULUCF sink is expected to decrease over time [38].

In this study we represent BAU development of net LULUCF emissions by the low sequestration scenario presented in the 6th National Communication [38]. The BAU scenario projects a reduction of the net LULUCF sink by about 339 Mt CO_2_e year^−1^ in 2030 (compared to 2010 level). The high sequestration scenario is on the other hand assumed to represent the INDC mitigation scenario development. Following the high sequestration scenario, the net LULUCF sink is expected to be increased by roughly 32 Mt CO_2_e year^−1^ in 2030, as compared to 2010 levels. Based on these assumptions, implementation of the INDC is expected to reduce net LULUCF emission by approximately 284 Mt CO_2_e year^−1^ in 2020 and 372 Mt CO_2_e year^−1^ in 2030, both compared to the BAU scenario.

The uncertainties for the BAU scenario from the USA LULUCF sector arise due to the absence of a BAU and an INDC mitigation scenario projection being mentioned in the INDC. It is assumed that the INDC target is consistent with the projections provided in the 6th National Communication, i.e. low and high sequestration scenarios. However, it remains uncertain which scenario can be considered as BAU or INDC mitigation scenarios.

Further Party specific details for Australia, Democratic Republic of the Congo (DRC), Ethiopia, Gabon, Japan, and Mexico are provided in the Additional file 1.

### Harmonization of scenarios to FAO historical estimates

In order to provide global coverage of net LULUCF emissions and comparability with other estimates, a harmonization factor was used to cover the net LULUCF emissions for Parties not analyzed in this study. Global emissions were thus harmonized to account for those Parties whose INDC does not address net LULUCF emissions and to provide historical greenhouse gas emissions. The global estimate of net LULUCF emissions from FAOSTAT [20] was used for this harmonization of emissions and removals according to the 2010 emission level of about 3 Gt CO_2_e year^−1^. The global harmonization factor was calculated as the difference between the global FAOSTAT estimate, and the sum of net LULUCF emissions derived for the 106 Parties that explicitly state in their INDC submission that the LULUCF sector is covered (see Table 1, two left columns). Hence, the harmonization factor covers the LULUCF emission and removals for the countries that are not considered in the INDC analysis, as well as differences between Parties’ reported net LULUCF emissions and the estimates reported by FAOSTAT. For the projections of global net LULUCF emissions, the harmonization factor is assumed to stay constant over time and thereby does not impact the global estimated emission reduction from the LULUCF sector, nor estimates for individual Parties.

A different approach to provide a global coverage of the LULUCF emissions and removals would have been to harmonize the 2010 level for each individual Party to that of the FAOSTAT estimate through the use of a Party specific harmonization factor. However, as such an approach would cause the Parties’ 2010 net LULUCF emissions to no longer be consistent with their respective projections of LULUCF emissions and removals, this approach was not used. If a Party specific harmonization factor had been used and projections of LULUCF emissions and removals had been scaled using the same harmonization factor, the same conclusions concerning Parties emission reduction from the LULUCF sector would have been drawn, if the global harmonization factor had been used. One difference that could have been observed is some Parties changing from between currently being net sources of LULUCF emissions, to being net removers of LULUCF emissions. However, such a change would not impact the estimated net mitigation related to implementation of the Parties’ INDC.

Further work on providing globally consistent estimates of historical LULUCF emissions and removals, in line with national inventory estimates, would help to reduce the fundamental uncertainty related to the harmonization factor.

## Additional file

**Additional file 1.** Additional material.

## Footnotes

1 In our analysis, the EU28 is considered as a single Party, and EU Member States are not considered individually. To facilitate reading, this paper uses ‘countries’ and ‘national’ to refer to all ‘Parties’, including the EU28.
