# Supplementary material for: Assessing the INDCs’ land use, land use change, and forest emission projections
Source: Carbon Balance Manag. 2016 Dec 8;11:26. doi: 10.1186/s13021-016-0068-3 (PMC5145905; doi:10.1186/s13021-016-0068-3)
Supplement: Supplementary file 1 — Additional file 1. Additional material. [file 13021_2016_68_MOESM1_ESM.docx]

## Supplementary Material

## Assessing the INDCs’ land use, land use change, and forest emission projections

##

Nicklas Forsell^[[1]](#footnote-2),*^, Olga Turkovska^1^, Mykola Gusti^[[2]](#footnote-3)^, Michel den Elzen^[[3]](#footnote-4)^, Michael Obersteiner^1^, Petr Havlik^1^

## Corresponding Author

[forsell@iiasa.ac.at](mailto:forsell@iiasa.ac.at)

## National baseline and INDC scenarios for Parties

Below follows a description of how the national business-as-usual (BAU) and INDC mitigation scenarios for the development of the LULUCF sector have been created for major Parties of interest.

**Australia**

The INDC of Australia does not provide explicit LULUCF emission projections corresponding to a BAU scenario nor an INDC mitigation scenario. Australia’s emission reduction target from the INDC is considered unconditional. However, the INDC report mentions Australia’s Emission Reduction Fund of the Australian Department of the Environment which “supports Australian businesses to reduce emissions while improving productivity” [[1](#_ENREF_1)]. According to the Australian Department of the Environment, net LULUCF emissions can be reduced by 28 Mt CO_2_e aggregated over the next 10 years through sequestration efforts supported by the Australia’s Emission Reduction Fund [[1](#_ENREF_1)]. Assuming a linear increase of the proposed mitigation efforts, it can be assumed that the annual reduction of net LULUCF emissions in 2025 will be approximately 5 Mt CO_2_e yr^-1^. No mitigation effort for the Australia’s Emission Reduction Fund has been provided for the period 2026 and 2030.

As Australia’s INDC does not report any projections for the BAU scenario, our BAU and INDC mitigations scenarios have been constructed based on the BAU scenario that was reported by Australia in the 6^th^ National Communication [[2](#_ENREF_2)] and Australia’s Emissions Projections 2012 Report [[3](#_ENREF_3)], updated according to 2015 UNFCCC reporting of the CRF tables. The reported BAU scenario assumes that the future deforestation rates will stay at the same level as the period 2008-2012 while reforestation removals will decline [[3](#_ENREF_3)]. Based on this, the BAU scenario shows a decrease of net annual LULUCF emissions by 2020 in the range of 3.6 Mt CO_2_e yr^-1^ compared to 2010 levels. As the BAU scenario has only been reported until 2020, it is assumed that net LULUCF emissions levels would remain constant in the BAU scenario over the period 2021 to 2030.

Historically, the 6^th^ National Communication [[2](#_ENREF_2)] and Australia’s Emissions Projections 2012 Report [[3](#_ENREF_3)] have provided LULUCF projections corresponding an BAU development and a scenario with measures. These estimates are here not considered. However, which projection that should be considered as the BAU for the INDC remains uncertain, given that both the BAU and scenario with measures presented in the Australia’s Emissions Projections 2012 Report could be considered as the BAU for the INDC given that some of the measures presented in the scenario with measures have been implemented.

It must be noted that in 2010 net LULUCF emissions in both the BAU and INDC mitigation scenarios have been updated according to 2015 UNFCCC reporting of the CRF tables. This has been done through the introduction of an emissions factor that remains constant over time. It should also be noted that neither the BAU nor the INDC mitigations scenario account for emissions from wildfires in line with the decision in the INDC to not account for emissions from wildfires.

**Democratic Republic of the Congo**

The INDC of the Democratic Republic of the Congo (DRC) covers the LULUCF sector, and also includes LULUCF emission projections up to 2030 corresponding to a BAU and INDC mitigation scenarios. The INDC target of the DRC is fully conditional, hence a fulfilment level of LULUCF sector objectives is undetermined. However, while LULUCF emissions are considered within the INDC, is appears that removals are excluded from historical estimates as well as the BAU projection. In the BAU scenario as provided by the INDC, LULUCF emissions increase from 193 Mt CO_2_e yr^-1^ in 2010 to roughly 400 Mt CO_2_e yr^-1^ in 2030. The INDC mitigation scenario foresees a rise of LULUCF emissions as well, but with slower growth rates than the BAU scenario. Under the INDC mitigation scenario, net LULUCF emissions are expected to increase from 193 Mt CO_2_e yr^-1^ in 2010 to roughly 300 Mt CO_2_e yr^-1^ in 2030. The INDC states that this reduction in LULUCF emissions will mainly be achieved through afforestation and reforestation measures.

**Ethiopia**

The INDC of Ethiopia provides LULUCF emission projections up to 2030, for both the BAU and INDC mitigation scenario. The mitigation measures proposed by the Ethiopian government include protection of forest areas, re-establishment of forests, and an increase of carbon stocks. Ethiopia’s INDC indicates that the net LULUCF emissions in 2010 were around 55 Mt CO_2_e yr^-1^. The BAU scenario assumes an increase of emissions over time and that the net LULUCF emissions would reach 90 Mt CO_2_e yr^-1^ in 2030, while the INDC scenario assumes a significant decline of emissions over time, bringing the net annual LULUCF emission to the level of -55 Mt CO_2_e yr^-1^ in 2030. According to the INDC, implementation of the measures is estimated to lead to emission reductions of approximately 65 Mt CO_2_e yr^-1^ in 2020 and 130 Mt CO_2_e yr^-1^ in 2030, both compared to net emissions as of the BAU scenario. If these reductions are indeed achieved, Ethiopia's LULUCF sector would become a net carbon sink by 2030, instead of a net source of emissions by 2010 in the BAU scenario. However, the INDC states that full implementation of the Ethiopia’s reduction target is possible with international support in terms of finance, infrastructure, technology, etc. Ethiopia published its 1^st^ National Communication in 2001 [[4](#_ENREF_4)] which provided historical level for net LULUCF emissions in 1994; however, no projections of LLULUCF sectorial emissions were reported.

**Gabon**

The data on emissions related to LULUCF sector in Gabon are limited. The 1^st^ National Communication of Gabon provides aggregated emission levels for agriculture and LULUCF in 1994 [[5](#_ENREF_5)], while in the 2^nd^ National Communication these emissions are reported separately for these two categories [[6](#_ENREF_6)]. Consequently, historical reporting on LULUCF emissions and removals are scarce for Gabon. The INDC of Gabon covers the LULUCF sector and also includes BAU emission projections for the LULUCF sector as well as the historical net LULUCF emissions for the year 2000. However, there is an inconsistency between historical net LULUCF emissions as reported in the INDC (80 Mt CO_2_e yr^-1^ in 2000) and historical net LULUCF emissions as reported in the 2^nd^ National Communication [[6](#_ENREF_6)] (-64 Mt CO_2_e yr^-1^ in 2000). This problem is discussed by the Climate Action Tracker [[7](#_ENREF_7)], and, to the extent of our knowledge, no clear explanation for the discrepancy has been presented yet.

The BAU and INDC mitigation scenario as provided in this study have been constructed based on the BAU scenario and data reported in the INDC. A few mitigation policies in the LULUCF sector are mentioned in the INDC (e.g., Code Forestier, Plan National d’Affectation de Terre). The mitigation potential for Gabon is expected to slightly increase over time, from 95 Mt CO_2_e yr^-1^ in 2020 to 102 Mt CO_2_e yr^-1^ in 2030, compared to the BAU scenario. The aforementioned data inconsistency is also assessed in terms of uncertainties; it is assumed that both scenarios may stay constant over time at the level of last available inventory data.

**Japan**

The INDC of Japan does not provide LULUCF projections corresponding to a BAU nor an INDC mitigation scenario. Projections for the LULUCF sector are also provided in the 6^th^ National Communication [[8](#_ENREF_8)]. Consequently, our BAU scenario conservatively assumes that LULUCF emissions and removals will stay constant over time, at the level of 2010, as updated according to 2015 UNFCCC reporting of CRF tables (-69 Mt CO_2_e yr^-1^). Japan’s INDC does provide an unconditional emission reduction target for the LULUCF sector by 2030, stating that net LULUCF emissions will be reduced by 37 Mt CO_2_eq yr^-1^ in 2030 compared to 2010 levels. This implies that the net LULUCF emissions could be reduced by 12 Mt CO_2_e yr^-1^ in the period 2010-2020. According to the INDC, approximately 75% of this reduction will be based on forest carbon sinks measures, while the remaining 25% will be the result of cropland management, grazing land management, and revegetation.

**Mexico**

The INDC of Mexico covers the LULUCF sector and also includes unconditional mitigation targets specifically for this sector (e.g. 0% deforestation by 2030). However, no LULUCF emission projections are provided corresponding to a BAU or INDC scenario. Therefore, our assessment uses the BAU scenario as reported in the 5^th^ National Communication [[9](#_ENREF_9)]. Overall, the BAU scenario assumes a 0.4% yearly decrease of net LULUCF emissions. Consequently, emissions will remain almost constant over the period 2010-2020 (around 46 Mt CO_2_e yr^-1^). A scenario with measures for the LULUCF is provided in the 5^th^ National Communication that shows a decline of the net LULUCF emissions level to 11 Mt CO_2_e yr^-1^ in 2020. Based on the quantification of Mexico’s Mitigation Portfolio as provided in the 5^th^ National Communication, Mexico can decrease net annual LULUC emissions by 35 Mt CO_2_e yr^-1^ in 2020, compared the BAU scenario. The 5^th^ National Communication does not provide a quantification of mitigation options for the LULUCF sector after 2020. If the estimated emission reductions would be achieved as specified, the LULUCF sector would still be a net source of emissions by 2020. However, with continued implementation of emission abatements options after 2020, the LULUCF sector could potentially become a net sink of emissions by 2030.

The source of uncertainties for Mexico’s future LULUCF emissions and removals is the BAU and INDC fulfilment projections after the year 2020. Both projection scenarios might be constant over time or continue to increase/decrease with the same rate as before 2020.

## References

1. Australian Government, *Australia's abatement task: tracking to 2020*. 2015, Department of the Environment, Australia: <http://www.environment.gov.au/climate-change/publications/factsheet-emissions-projections-2015-16>.

2. Government of Australia, *Australia’s Sixth National Communication on Climate Change – A report under the United Nations Framework Convention on Climate Change. Canberra: Department of Industry, Innovation, Climate Change, Science, Research and Tertiary Education*. 2013.

3. Government of Australia, *Australia's emissions projections 2012*. 2012, Department of Climate Change and Energy Efficiency, Australia: <http://www.climatechange.gov.au/publications/projections/australias-emissions-projections.aspx>.

4. Government of Ethiopia. *Initial national communication of Ethiopia to the United Nations Framework Convention on Climate Change,* [*http://unfccc.int/resource/docs/natc/ethnc1.pdf*](http://unfccc.int/resource/docs/natc/ethnc1.pdf). 2001.

5. Government of Gabon. *Ministère de l'environnement et de la protection de la nature. Direction de l'environnement. Communication nationale sur les changements climatiques,* [*http://unfccc.int/resource/docs/natc/gabnc1.pdf*](http://unfccc.int/resource/docs/natc/gabnc1.pdf). 2004.

6. Government of Gabon. *MINISTERE DE L’HABITAT, DE L’URBANISME, DE L’ECOLOGIE ET DU DEVELOPPEMENT DURABLE. Seconde Communication Nationale du Gabon sur le Changements,* [*http://unfccc.int/resource/docs/natc/gabnc2.pdf*](http://unfccc.int/resource/docs/natc/gabnc2.pdf). 2011.

7. Climate Action Tracker, [*http://climateactiontracker.org/*](http://climateactiontracker.org/). 2015.

8. Government of Japan, *Sixth National Communication to the United Nations Framework Convention on Climate Change,* [*http://unfccc.int/files/national_reports/annex_i_natcom/submitted_natcom/application/pdf/nc6_jpn_resubmission.pdf*](http://unfccc.int/files/national_reports/annex_i_natcom/submitted_natcom/application/pdf/nc6_jpn_resubmission.pdf). 2012.

9. Government of Mexico, *Mexico. México quinta comunicación nacional ante la Convención Marco de las Naciones Unidas sobre el Cambio Climático.* 2012, <http://unfccc.int/resource/docs/natc/mexnc5s.pdf>.

1. International Institute for Applied System Analysis (IIASA), Schlossplatz 1, 2361 Laxenburg, Austria [↑](#footnote-ref-2)
2. Lviv Polytechnic National University, 12 Bandera street, 79013 Lviv, Ukraine [↑](#footnote-ref-3)
3. PBL Netherlands Environmental Assessment Agency, Bezuidenhoutseweg 30, 2594 AV, The Hague [↑](#footnote-ref-4)
